# Current concepts in clinical radiation oncology

**DOI:** 10.1007/s00411-013-0497-2

**Published:** 2013-10-20

**Authors:** Michael Orth, Kirsten Lauber, Maximilian Niyazi, Anna A. Friedl, Minglun Li, Cornelius Maihöfer, Lars Schüttrumpf, Anne Ernst, Olivier M. Niemöller, Claus Belka

**Affiliations:** 1 Department of Radiotherapy and Radiation Oncology, Ludwig-Maximilians-University of Munich, Munich, Germany; 2 Present Address: Clinic for Radiation Oncology, St. Elisabeth Hospital Ravensburg, Ravensburg, Germany

**Keywords:** Radiotherapy, IMRT/IGRT, Particle therapy, Targeted therapy, Biomarkers, Personalized medicine

## Abstract

Based on its potent capacity to induce tumor cell death and to abrogate clonogenic survival, radiotherapy is a key part of multimodal cancer treatment approaches. Numerous clinical trials have documented the clear correlation between improved local control and increased overall survival. However, despite all progress, the efficacy of radiation-based treatment approaches is still limited by different technological, biological, and clinical constraints. In principle, the following major issues can be distinguished: (1) The intrinsic radiation resistance of several tumors is higher than that of the surrounding normal tissue, (2) the true patho-anatomical borders of tumors or areas at risk are not perfectly identifiable, **(**3) the treatment volume cannot be adjusted properly during a given treatment series, and (4) the individual heterogeneity in terms of tumor and normal tissue responses toward irradiation is immense. At present, research efforts in radiation oncology follow three major tracks, in order to address these limitations: (1) implementation of molecularly targeted agents and ‘omics’-based screening and stratification procedures, (2) improvement of treatment planning, imaging, and accuracy of dose application, and (3) clinical implementation of other types of radiation, including protons and heavy ions. Several of these strategies have already revealed promising improvements with regard to clinical outcome. Nevertheless, many open questions remain with individualization of treatment approaches being a key problem. In the present review, the current status of radiation-based cancer treatment with particular focus on novel aspects and developments that will influence the field of radiation oncology in the near future is summarized and discussed.

## Introduction

Cancer is the second most frequent cause of death within developed countries being responsible for 200–400 deaths per 100,000 people each year. The incidence of cancer is closely related to age, indicating that the probability of malignant transformation increases with life span. Additionally, cancer can evolve due to risk factors, such as cancer-causing lifestyle habits (e.g., cigarette smoking), genetic predisposition, and viral infections.

Radiotherapy, the clinical application of ionizing radiation, is one crucial treatment option in modern cancer therapy apart from surgery and systemic therapy as being corroborated by the fact that more than 60 % of all cancer patients receive radiotherapy today. Radiotherapy can be used in various treatment settings ranging from definitive strategies to multimodal settings, e.g., in adjuvant and in neoadjuvant settings, with or without concomitant chemotherapy. The efficacy of radiotherapy has been proven in multiple randomized trials and has been described in meta-analyses that included multiple cancer types. Radiotherapy can significantly prolong patient survival and improve the local control rates of tumors. Furthermore, radiotherapy can help to avoid surgical amputation and to yield better cosmesis, and it can be used in palliative settings (Ringborg et al. 2003; Delaney et al. 2005).

For the treatment of head and neck cancer, radiotherapy may be used postoperatively, e.g., for patients with specific risk factors (Bernier et al. 2004; Cooper et al. 2004), but it has also been proven to be effective as primary definitive treatment strategy—particularly when being combined with concomitant chemotherapy (Pignon et al. 2009). In case of lung cancer, radiotherapy can be applied stereotactically for the treatment of early forms of bronchial carcinoma achieving high rates in local control (Guckenberger et al. 2009; Timmerman et al. 2010), and for advanced stages, it can be used in a neoadjuvant, adjuvant, or definitive manner as well as for palliation, respectively (Auperin et al. 2010; Albain et al. 2009; Douillard et al. 2006). For breast cancer, it was shown that breast-conserving surgery in combination with adjuvant radiotherapy results in survival rates that are equal to mastectomy (Fisher et al. 2002) and that omitting adjuvant radiotherapy causes a decrease by 4 % in patient survival (Darby et al. 2011). Finally, in case of prostate cancer, radiotherapy with or without combined hormone therapy reveals comparable cure rates as surgical treatment efforts (Bolla et al. 2002), albeit randomized trials are missing. Taken together, all these findings demonstrate the importance of radiotherapy as one of today’s crucial cancer treatment strategies, and the evidence for its effectiveness is still expanding.

## Technical improvements in precision of radiotherapy

**Fig. 1:**
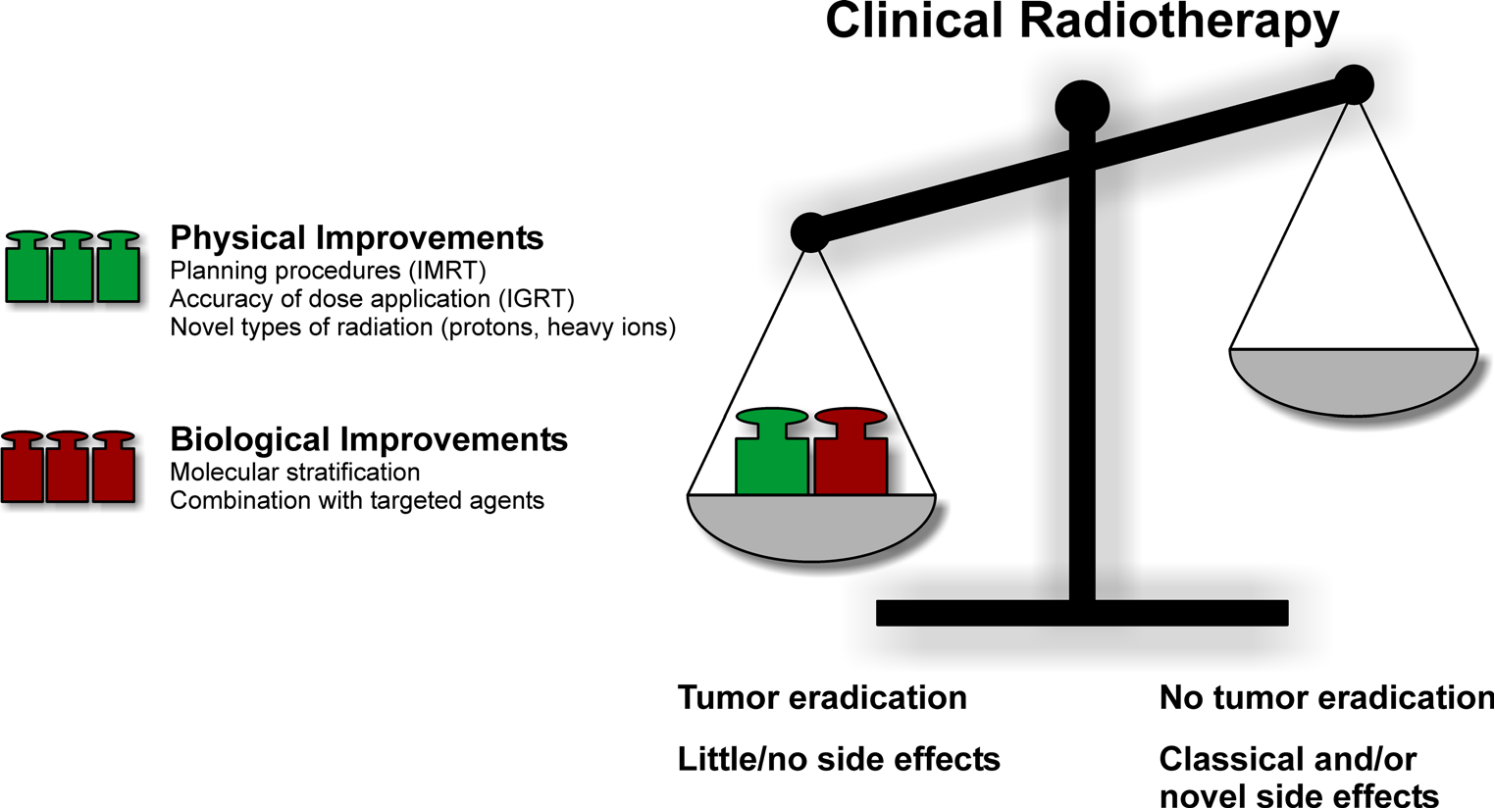
Improvements in clinical radiotherapy with decisive impact in recent years

Since ionizing radiation is extremely effective in killing any kind of eukaryotic cell, a relevant therapeutic gain is only obtained when several prerequisites are met: adequate fractionation, optimal target delineation, radiation planning, image guidance, and toxicity diversification (radiochemotherapy). In recent years, intensity-modulated radiotherapy (IMRT) and image-guided radiotherapy (IGRT) comprise the most important technological advances (Fig. 1).

### Intensity-modulated radiotherapy (IMRT) and image-guided radiotherapy (IGRT)

In principle, all radiation techniques that employ a non-homogenous photon fluence over a given radiation field can be considered as “intensity modulated.” In a more narrow sense, IMRT describes the sequential accumulation of multiple radiation fields resulting in a non-homogenous photon fluence from different gantry angles (Glatstein 2002). Currently, several variations in the IMRT principle are being used to achieve highly conformal radiation distributions: Classical IMRT, volumetric-modulated arc therapy (VMAT), Rapid Arc^®^, Tomotherapy, and Cyberknife are different technological/vendor-specific solutions that are used to achieve optimal dose distributions while sparing normal tissues in an optimal fashion. To date, it has been proven that the use of IMRT achieves better conformity of the high-dose region to the target volume when compared to 3D conformal approaches, especially for complex treatment situations (Bortfeld 1999), in which adjacent organs at risk might compromise full coverage of the target volume (Mok et al. 2011).

Up to now, many trials have been performed documenting the feasibility of increased target doses with reduced toxicity using IMRT. Probably, the best examples are sparing of the parotid gland in head and neck cancer (Hey et al. 2011) and sparing of the rectum and the bladder while concomitantly increasing the target dose in prostate cancer (Takeda et al. 2012). However, randomized data that compare IMRT with classical 3D conformal radiotherapy are rare (Gupta et al. 2012). This is clearly related to the fact that it is difficult to set up a randomized trial whenever obvious differences in high-dose distributions are visible already after radiation plan comparison. In the meantime, more advanced rotational IMRT techniques such as VMAT and RapidArc^®^ have entered clinical practice and allow for even faster application of prescribed doses. However, the clinical benefits of these techniques need to be further investigated (Jiang et al. 2011; Wiezorek et al. 2011; Foroudi et al. 2012; Fogarty et al. 2011). At present, the development strategies in the field of IMRT and related techniques basically aim at further improving the underlying planning and optimization algorithms as well as the technology of the LINACs in use. However, several open issues are not yet fully solved: (1) dose optimization in case of non-homogenous dose distributions, (2) toxicity prediction in case of non-homogenous doses to organs at risk, (3) reproducibility and verification of treatments with strongly increasing degrees of freedom (rotation, rotation speed, dose rate, field shape, etc.), and (4) mechanical stability and reliability of all components in use. Therefore, one focus of research is the development of planning algorithms, including tools for biological optimization and improved dose calculation (Monte Carlo calculations or similar). In addition, the technology providers aim at developing LINACs that are more and more “ab initio” designed for the implementation of the technologies mentioned above. In parallel, with increasing precision of radiation planning and dose application, the need for better target acquisition raises strongly. The term “target acquisition” covers merely all aspects of patient positioning, patient movement, internal organ movement between fractions, and internal organ motion within a fraction. In this regard, many different visualization tools are in use or in clinical testing. IGRT tools range from classical electronic portal imaging devices (EPIDs) (Njeh et al. 2012) and MV and kV cone-beam CTs (Foster et al. 2012) to complex 3D ultrasound (Chadha et al. 2011) and surface scanners (Pallotta et al. 2012). The wide use of these imaging devices will change the classical target volume approaches considerably. To date, the gross target volume (GTV) -> clinical target volume (CTV) -> planning target volume (PTV) concept is rather static using predefined safety margins in order to compensate for any kind of movement. Replacing this paradigm by daily “online” controls allows for smaller margins, which only reflect biological uncertainties.

In a wider sense, the term “IGRT” describes the use of advanced imaging technology, in order to optimally define target volume sites and organs at risk. At present, several imaging modalities have entered clinical practice in radiation oncology. 18F-Fluorodeoxyglucose (FDG)-based positron emission tomography (PET)-CT is frequently helpful during target volume definition. Highly specific PET markers such as tetraazacyclo-dodecane-tetraacetic acid (DOTA)-octreotide (DOTATOC) and DOTA-octreotate (DOTATATE) strongly improve the definition of the target volume for meningioma (Gehler et al. 2009). Even less specific markers (e.g., 18F-fluoroethyltyrosine (FET)-PET) may strongly influence radiation treatment planning for glioma patients. In this regard, several groups have shown that FET-PET alters treatment volumes in roughly 50 % of the cases (Niyazi et al. 2012b). Nevertheless, many issues are currently unsolved: Specificity and sensitivity of merely all tracers are not high enough to allow for automated segmentation of the target volumes. Besides PET-CT, other means of advanced imaging also influence target volume delineation in radiation oncology. At present, the definition of the adjuvant lymphatic drainage region follows empiric and pragmatic rules (Vorwerk and Hess 2011) rather than individual patient-oriented considerations. For the prostate, several groups have analyzed the feasibility of SPECT-based sentinel analysis to define individual lymphatic regions at risk (Vees et al. 2012; Ganswindt et al. 2007). Similarly, more specific MRI tracers would be of key importance for improved target volume definition in various disease sites (Weidner et al. 2011). Thus, it is clear that the combination of improved imaging, both for delineation of the target volume and during treatment, will play a key role in future radiation oncology (Xing et al. 2006). In this regard, the use of IGRT results already today in less acute toxicity during radiotherapy, e.g. in case of prostate cancer (Gill et al. 2011; Crehange et al. 2012).

### Protons and heavy ions

Several recent developments like IMRT allow for the reduction in the dose exposed to normal tissue while keeping the prescribed dose on the tumor volume. However, these methods come at the cost of increasing the volume of normal tissues receiving low or moderate doses, and it has been assumed that this may increase the risk of radiation-induced secondary cancers (Hall 2009) although clinical or epidemiological data are not available yet. Charged particles such as protons or heavy ions deliver the highest dose near the end of their range, in the so-called Bragg peak. This allows for extremely steep dose gradients distal to the Bragg peak and thus for superior sparing of organs at risk in the vicinity of the target. Because there is, apart from a dose that is due to secondary particles or fragments, no exit dose and because entrance doses are lower than in the case of photons, this allows for an overall reduction in the integral dose outside the planned target area, which is expected to significantly reduce the risk of radiogenic secondary malignancies in long-term cancer survivors (Fontenot et al. 2010; Newhauser and Durante 2011). So far, no long-term epidemiological studies on the incidence of secondary cancer cases following a proton- or a heavy ion-based cancer treatment are available, and given the latency period associated with radiation-induced tumors, these studies will also not be available in nearer future. The knowledge of radiation-induced tumorigenesis and the many parameters involved (e.g., radiation dose and quality, fractionation, age at exposure, genetic susceptibility) is limited, and therefore, risk estimations are difficult to perform. For example, passive beam scattering, which has been the predominant method for increasing the size of the proton pencil beam generated by the accelerator up to now, produces secondary neutrons with a broad range of energies for some of which the relative biological effectiveness (RBE) is poorly characterized (Hall 2009), and therefore, the impact of these neutrons on secondary tumor risk is difficult to estimate. It should be noted that part of secondary neutron production is reduced in particle therapy setups using active beam scanning (Clasie et al. 2010).

So far, only a few clinical studies have been performed on the efficacy and acute side effects of proton and ion therapy, and only very few of them have directly compared the outcome of particle therapy and conventional radiotherapy. Brada et al. (2009) gave a detailed overview on the clinical impact of proton therapy based on a search within published, peer-reviewed literature. They identified 52 studies of proton therapy fulfilling their quality criteria (at least 20 patients with a follow-up period of at least 2 years), encompassing data of in total 13,736 patients (Brada et al. 2009). Of these patients, 10,328 received treatments for ocular tumors and 1,642 were treated for prostate tumors and 880 for tumors of the central nervous system (CNS). Other tumor entities such as head and neck tumors, gastrointestinal tumors, lung cancer, and sarcomas were subjects of two to five studies each, encompassing between 97 and 375 patients per tumor site. This number must be compared to more than 60,000 patients who had undergone a proton-based cancer therapy by the end of 2008 (http://ptcog.web.psi.ch/Archive/Patientstatistics-update02Mar2009.pdf). Brada and coauthors concluded that the evaluated literature lacks any evidence demonstrating a clear benefit of proton-based therapy if compared to the best available conventional therapies with respect to tumor control, patient survival, and side effects. Others studies came to similar conclusions, even with respect to pediatric tumors (Bouyon-Monteau et al. 2010), prostate cancer (Kagan and Schulz 2010), lung cancer (Liao et al. 2011), head and neck cancers (Ramaekers et al. 2011), and tumors of the skull base treated by radiosurgery (Amichetti et al. 2012). A recent study even showed higher rates of gastrointestinal side effects after a proton-based therapy if compared to conventional IMRT of prostate cancer (Sheets et al. 2012), but the methodology applied in this study is under debate (Deville et al. 2012; Mendenhall et al. 2012; Jacobs et al. 2012). Clearly, the absence of evidence is not evidence of absence of a superior efficacy or tolerance of proton therapy, but nevertheless, these analyses clearly stress the requirement of more clinical studies assessing the clinical impact of proton-based cancer therapy.

The better the conformity, the higher are the requirements for setup reproducibility, accuracy in patient immobilization, and consideration of changes in the patient’s anatomy, such as the motion of organs (e.g., due to filling of the bladder or the rectum), or treatment-induced alterations, e.g., tumor shrinkage. This holds for a highly conformal therapy with both photons and protons. The impact of intrafraction mobility, which is affected by the duration of the treatment, may be of special importance in case of an active proton beam scanning, because this method takes considerably more time than passive scattering or photon irradiation. Importantly, in the case of protons, an additional level of complexity comes into play since absorption and scattering of protons largely depend on the material traversed so that the range and the lateral penumbra are affected by the inhomogeneity of the tissue. Uncertainty in estimating the particle range will automatically translate into dose uncertainties. In spite of demands for state-of-the-art imaging, image guidance, and dose verification, several authors raised concerns about the lack of optimal technologies at proton therapy facilities (Merchant 2009; Schippers and Lomax 2011). As already pointed out by Goitein in 2008, the possibility for treatment errors is much greater in case of protons than with photons and therefore, proton therapy has to be used exclusively in a highly controlled fashion (Goitein 2008).

Carbon ions are less affected by energy straggling and scattering as compared to protons, and therefore, the precision of the dose deposition achievable is even greater than in the case of protons. However, due to fragmentation processes, a dose tail is always present distally from the Bragg peak, which must be considered in treatment planning. These fragmentation processes come, however, also with an advantage, namely the generation of positron emitters that allow for in situ beam monitoring (Weber and Kraft 2009). One major potential of carbon ions lies in the fact that they can confer a significant higher RBE than photons within their Bragg peak region, and this not only means that the physical dose there is highest, but also the biological effect achievable per dose unit. The expenses for carbon-ion-based radiotherapy units are, however, even greater than for proton facilities, and only few facilities have been available in the past. Since 2009, the carbon ion radiotherapy unit at the Heidelberg Ion Therapy (HIT) center which uses active beam scanning is operating, and initial data on clinical experiences become available now (Combs et al. 2010b). At HIT, all patients are treated within clinical trials (Combs et al. 2010a, c; Jensen et al. 2011a, b), and recently, randomized phase III trials have been initiated to compare proton- and carbon-ion-based therapies for the treatment of chondrosarcomas and chordomas (Nikoghosyan et al. 2010a, b). Due to their higher RBE, the treatment with carbon ions might be more effective for the cure of radioresistant tumors. A recent meta-analysis performed in different head and neck cancers compared the efficacies of photons, protons, and carbon ions (Ramaekers et al. 2011) but, so far, only revealed a survival benefit for mucosal malignant melanomas after a carbon-ion-based therapy, which might reflect a high grade of resistance of this particular tumor entity toward irradiation in general. Other work suggests that due to the reduced volume of normal tissue that is exposed to modest doses, particle therapy may confer advantages in treatments using concurrent drug administration (Nystrom 2010). In a modeling study, Vogelius et al. (2011) estimated the pneumonitis risk after a treatment with photons or protons either in combination with or in the absence of chemotherapy and came to the conclusion that proton therapy could potentially minimize the risk by reducing the volume that is exposed to lower doses (Vogelius et al. 2011). Given the increasing role of multimodality treatment approaches, further investigations into the relative merit of particle therapy in these settings are clearly needed.

The controversial discussion on the necessity of clinical studies of particle therapy is, in part, fuelled by the high costs of this treatment if compared to established photon therapy. One part of such elevated costs is due to the size of the synchrotrons or cyclotrons used, and there are several developments that aim for provision of smaller accelerators (Schippers and Lomax 2011). One putative solution could be the acceleration of protons and also of heavier ions by laser acceleration (Tajima 2010). Although current technologies are far from clinical application, some research groups already started to address the question of whether the RBE of laser-driven particles may differ from that of conventionally accelerated particles, thereby focusing on the ultrashort pulsing process by which these particles are generated as well as on the ultra-high dose rates associated with it (Rigaud et al. 2010; Yogo et al. 2009; Kraft et al. 2010; Bin et al. 2012). By simulating the pulsed radiation conditions expected in therapy settings using laser-accelerated protons of a pulsed proton beam at the Munich ion microbeam SNAKE (Dollinger et al. 2009), an extensive series of experiments with various endpoints in cell monolayers, 3D tissue culture models, and tumor xenografts were conducted. However, no significant differences between a dose of a few Gy that was given in about 1 ns (the dose rate expected after laser acceleration) and the same dose given in about 100 ms (the dose rate at conventional irradiation settings) could be observed in these experiments (Schmid et al. 2009, 2010; Auer et al. 2011; Greubel et al. 2011; Zlobinskaya et al. 2012).

## Biological improvements of radiotherapy

During the last decades, significant improvements have been made: A special focus has been placed on the development of advanced planning procedures (van Herk 2004), the physical accuracy of dose application (Bucci et al. 2005) and combined modality treatment approaches in terms of radiochemotherapy (Al-Sarraf et al. 1998) (Fig. 1). However, dose escalation studies revealed that the combination of radiotherapy with classical chemotherapy has reached some kind of dead end (Budach et al. 2006). At this point, the combination of radiotherapy with molecularly designed agents specifically targeting the hallmarks of cancer has revealed significant improvements in clinical outcomes when compared to each treatment strategy alone (Begg et al. 2011). However, the effective integration of molecularly targeted drugs requires a detailed patient stratification, since only those patients with relevant signal aberrations will benefit. Furthermore, it has to be noted that stratification is urgently needed in order to avoid side effects induced by the addition of such targeted drugs (Niyazi et al. 2011b). In the following paragraphs, the key biological targets for specifically improved radiotherapy will be introduced.

### The hallmarks of cancer

The emergence of cancer, in general, is due to failures within mechanisms or pathways that control the growth, the proliferation, and/or the death of cells in response to extracellular or intracellular signals. Deregulations within these mechanisms can commit cells to sustained proliferation, replicative immortality, evasion of growth suppression, and resistance to cell death—attributes commonly shared by malignantly transformed cells (Hanahan and Weinberg 2000). However, the transition from a single transformed cell toward the formation of a solid tumor requires additional features, such as the capacity to instigate the formation of blood vessels (angiogenesis and/or neovascularization), mechanisms to evade immune responses, as well as an increased potential to invade other tissues (metastasis) (Hanahan and Weinberg 2011).

#### Sustained proliferation and replicative immortality

The growth as well as the proliferation of cells is orchestrated by a class of signaling molecules called mitogens. While in non-transformed cells, the synthesis and the release of mitogens are tightly controlled, these processes are often deregulated in cancer cells. Such deregulation can be due to the acquisition of genetic mutations (for instance due to exposure to tumor-initiating chemicals and/or ionizing radiation) or to the experience of growth-supporting signals, such as tumor-promoting chemicals and chronic inflammation. Two of the best-characterized mitogens are the platelet-derived growth factor (PDGF) and the epidermal growth factor (EGF). The binding of these ligands to their respective receptors, PDGFR and epidermal growth factor receptor (EGFR), activates sophisticated signaling pathways, including the mitogen-activated kinase (MAP kinase) pathway, thereby stimulating both the growth and the proliferation of cells (Seger and Krebs 1995). Mutations within the genes that encode for such mitogens/receptors can render the corresponding gene products in a state of constitutive activation culminating in uncontrolled growth and/or proliferation of cells. In this regard, the gene encoding the small GTPase K-Ras provides a prototypical example as activating mutations of K-Ras are found in diverse cancer entities, e.g., in more than 40 % of all colorectal cancers (Karapetis et al. 2008). Similar examples can be found in other mitogenic signaling pathways, including the phospho-inositide-3-kinase (PI3 K)/AKT kinase and the insulin-like growth factor (IGF) pathway (Chang et al. 2003; Fresno Vara et al. 2004; Samani et al. 2007; Frasca et al. 2008).

With regard to their impact on the outcome of radiotherapy, both overexpression and mutation of EGFR were shown to correlate with increased resistance of tumors to irradiation and poor clinical prognosis (Lammering et al. 2003, 2004; Giralt et al. 2005; Milas et al. 2004). Furthermore, ligand-independent activation of EGFR in response to irradiation and the subsequent activation of its downstream signaling cascades apparently contribute to radioresistance (Iyer et al. 2004; Gupta et al. 2001; Toulany et al. 2005). Therefore, multiple strategies have been developed in order to interfere with EGFR function as being discussed in more detail later on.

A key step in malignant transformation is the acquirement of basically limitless replicative potential. After a certain number of division cycles, a normal cell exits the cell cycle and transits into senescence, a stage of metabolic activity devoid of further proliferation (Campisi and d’Adda di Fagagna 2007). The induction of senescence requires a group of proteins encoded by genes that are known as tumor-suppressor genes (e.g., p53, pRB). These genes negatively regulate the growth and/or the proliferation of cells, and hence, mutations that render their products inactive can support both immortalization and unrestrained proliferation. Another prerequisite for replicative immortality is the cell’s capacity to protect its telomeres (Blasco 2005). Since expression of telomerase is almost absent in non-immortalized cells, their replicative potential is greatly limited by successive telomere shortening. In immortalized cells (including cancer cells), to the contrary, expression of telomerase is reinitiated, thereby counteracting the erosion of telomeres and, in consequence, the induction of senescence or apoptosis. Additionally, expression of telomerase and telomere length have been reported to contribute to radioresistance of tumor cells (Genesca et al. 2006).

#### Evasion of growth suppression and resistance to cell death

Aside from extensive proliferation, the formation of solid tumors necessitates the cellular capacity for evading growth-suppressive signals, which mostly depend on tumor-suppressor proteins, such as p53 or the members of the retinoblastoma protein family. These proteins interfere with cell proliferation in response to growth-inhibiting signals and/or intracellular disorders including DNA damage either by blocking the expression of genes required for cell cycle progression or by initiating the expression of cell cycle-inhibiting genes such as p16^INK4a^ and p21^WAF1^ (Sherr and Roberts 1999). Alternatively, tumor-suppressor proteins (in particular p53) can also stimulate the induction of a programmed form of cell death called apoptosis, e.g., in response to DNA damage, explaining p53’s pivotal role in determination of tumor radiosensitivity (Gudkov and Komarova 2003). In this context, p53 induces the expression of several pro-apoptotic proteins (e.g., PUMA) and thereby facilitates the induction of apoptosis. However, many cancer cells circumvent apoptosis, e.g., by inactivating p53, by down-regulating pro-apoptotic genes, or by up-regulating antiapoptotic genes.

#### Angiogenesis and neovascularization

Since the formation of solid tumors demands for a continuous nutrient and oxygen supply, tumor cells must acquire the capacity to stimulate vascularization involving de novo formation of blood vessels (vasculogenesis) as well as sprouting of newly formed vessels from preexisting ones (angiogenesis). In adults, angiogenesis and vasculogenesis are tightly limited to certain physiological processes, such as wound healing. However, during tumor progression, angiogenesis is reinitiated (Bergers and Benjamin 2003). To this end, tumor cells secrete pro-angiogenic factors, such as the vascular endothelial growth factor (VEGF) that, upon binding to their respective receptors (VEGFR), stimulate the proliferation of endothelial cells resulting in increased vessel formation and tumor infiltration. VEGF expression in tumor cells is facilitated by certain oncogene products, including c-Myc or H-Ras, whereas non-transformed cells express VEGF almost exclusively under hypoxic conditions (Baudino et al. 2002; Chin et al. 1999). The degree of vascularization plays an important role in regard to the tumor’s responsiveness to ionizing radiation. As the induction of DNA damage is supported by the presence of oxygen, increased hypoxia limits the efficacy of radiotherapy. Consequently, intense efforts are spent in order to increase tumor oxygenation and to improve the therapeutic effect of exposure to ionizing radiation (Wachsberger et al. 2003).

#### Evasion of immune responses

Another barrier limiting the formation and the progression of tumors is the immune system. This becomes clear by the fact that immunocompromised mice, e.g., mice that are deficient in CD8^+^ T lymphocytes or natural killer (NK) cells, show a significant higher susceptibility to cancer than those that are immunocompetent (Schreiber et al. 2011). Consequently, it is no wonder that tumor cells acquire multiple mechanisms to evade immune responses, such as elimination and/or aberration of tumor antigens/MHC class I molecules, secretion of immunosuppressive cytokines such as transforming growth factor β (TGF-ß) and interleukins, recruitment of immunosuppressive immune cells (e.g., CD4^+^ CD25^+^ regulatory T cells and myeloid-derived suppressor cells), or expression of indolamine-2,3-dioxygenase (IDO) (Kaufman and Disis 2004; Munn and Mellor 2007; Garcia-Lora et al. 2003). Several lines of evidence support the notion that the immune system plays a pivotal role in tumor regression in response to radiotherapy (Lauber et al. 2012). This is of particular interest, since the induction of an antitumor immune response might not only be helpful for the elimination of the primary tumor within the irradiation field, but also for out-of-field metastases (Frey et al. 2012).

#### Tissue invasion and metastasis

Aside from their capacity to form primary tumors, some malignantly transformed cells also acquire the capacity to infiltrate neighboring tissues or even penetrate lymphatic and/or blood vessels, giving rise to several kinds of secondary tumors or metastases. Usually, metastasis starts with the detachment of tumor cells from the primary tumor site facilitated by the repression of factors that mediate cellular adhesion, such as E-cadherin, and by secretion of enzymes that degrade extracellular matrices (ECMs), thus liberating tumor cells from their surroundings (Valastyan and Weinberg 2011). These processes depend on the activation of a conserved cellular program termed the epithelial–mesenchymal transition (EMT), which regulates the formation of the mesoderm and the neural tube during embryonic development (Thiery et al. 2009). For several tumor entities, glioblastomas in particular, it was shown that irradiation increases their invasive potential and thus might even accelerate local dissemination and development of distant metastasis (Qian et al. 2002; Cordes et al. 2003; Wild-Bode et al. 2001; Camphausen et al. 2001).

### Mechanisms of cell death

**Fig. 2:**
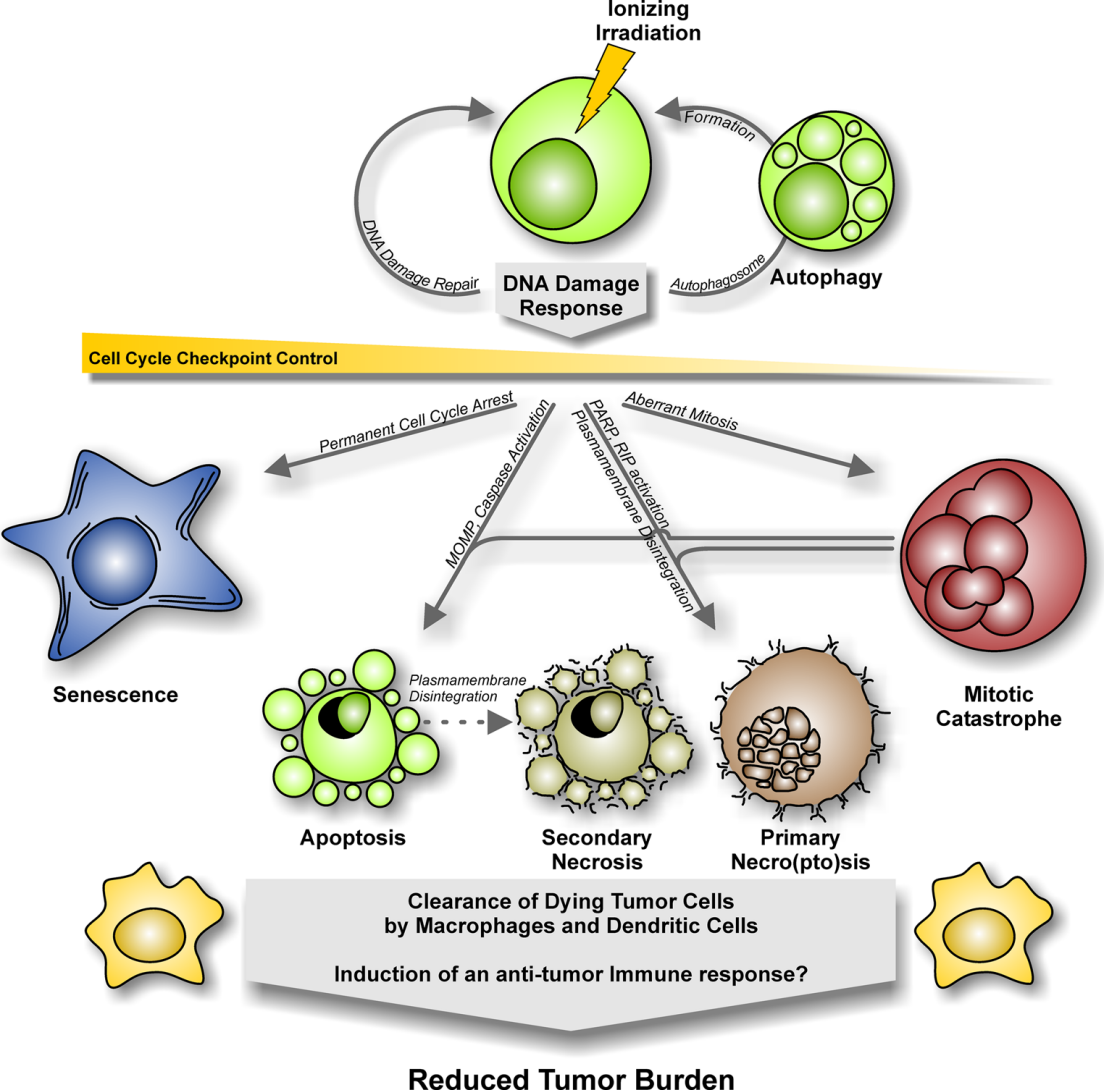
Mechanisms of cell death triggered by ionizing radiation

Radiotherapy is an important treatment modality in clinical cancer therapy because of its great potential to kill malignant cells and to abrogate clonogenic survival. Directly or indirectly, ionizing radiation induces different types of genome damage, including DNA double-strand breaks (DSBs), bulky lesions, and others, thereby activating a highly sophisticated signaling network termed the DNA damage response (DDR) culminating in transient or permanent cell cycle arrest and/or cell death, respectively (Fig. 2).

#### DNA damage response (DDR)

The DDR mediates cellular responses to various kinds of DNA damage, a cell has to cope with. The DDR is regulated by two conserved protein kinases called Ataxia telangiectasia mutated (ATM) and Ataxia telangiectasia and Rad3 related (ATR) (Smith et al. 2010). ATM is recruited to DSBs by the Mre11-Rad50-Nbs1 (MRN) complex where it phosphorylates the histone H2 variant H2AX, thereby creating a recruitment platform for other DDR factors (Shiloh 2006). In parallel, ATM mediates resection of the broken DNA strand(s), and the resulting ssDNA repair intermediates specifically activate ATR kinase (Hurley and Bunz 2007). By phosphorylation of two respective downstream kinases termed CHK1 and CHK2, ATR and ATM trigger a multitude of signaling pathways, thereby initiating both a transient arrest within cell cycle progression and DNA damage repair. However, in case of excessive DNA damage, ATM/ATR can also induce cellular senescence and/or cell death (Jackson and Bartek 2009).

The major target of the ATM/ATR cascade in terms of arresting the cell cycle or committing the cell to cell death is the tumor-suppressor protein p53. In the absence of DNA damage, the overall levels of p53 within the cell are maintained rather low because of the association of p53 with the ubiquitin ligase MDM2 (HDM2 in human). MDM2 continuously ubiquitylates p53, thereby targeting p53 for proteasomal degradation. Yet, in the context of the DDR, p53 is phosphorylated by kinases of the ATM/ATR cascade leading to its dissociation from MDM2 and thus to stabilization of p53 (Meek 2009). Once being stabilized, the transcription factor p53 crucially regulates cell cycle arrest, DNA damage repair, and the induction of cell death or senescence by inducing or repressing the expression of several target genes that encode for factors involved in these processes (Sengupta and Harris 2005).

#### Apoptosis

Apoptosis is a form of programmed cell death, which is characterized by chromatin condensation/fragmentation, cell shrinkage, and blebbing of cell membranes. In response to irradiation, apoptosis is predominantly observed in cells of the hematopoietic system. Radiation-dependent induction of apoptosis mainly relies on the intrinsic death pathway (Rudner 2001), in which cytochrome c is released into the cytosol by permeabilization of the outer mitochondrial membrane. This, in turn, stimulates the formation of the apoptosome and subsequent activation of the caspase cascade. The cleavage of multiple caspase substrates within the cell finally results in chromatin fragmentation, organelle destruction, and cellular disintegration (Taylor et al. 2008). The process of mitochondrial permeabilization is essentially controlled by pro- and antiapoptotic members of the B-cell lymphoma-2 (Bcl-2) family, which regulate the channel-forming activity of the family members BAX and BAK (Chipuk et al. 2010; Youle and Strasser 2008). Protein p53 can modulate this equilibrium in response to DNA damage by inducing the expression of pro-apoptotic Bcl-2 family members, such as PUMA, NOXA, and BAX itself (Sengupta and Harris 2005).

Stimulation of apoptosis via the extrinsic death pathway, on the contrary, depends on the binding of death ligands (e.g., CD95L, TRAIL) to their respective cell surface receptors (Debatin and Krammer 2004). Subsequent death receptor clustering triggers the activation of the caspase cascade in this pathway. Although the expression levels of several key regulators of the extrinsic pathway have been described to increase upon exposure to ionizing radiation (Belka et al. 1998; Haupt et al. 2003), the intrinsic pathway appears to be the dominant pathway of apoptosis induction in response to DNA damage (Rudner 2001). Additionally, it should be noted that cells deficient in p53 function can undergo radiation-induced apoptosis as well, indicating that alternative mechanisms such as p63-/p73-dependent expression of pro-apoptotic factors can compensate for the lack of p53 in these cases (Afshar et al. 2006; Wakatsuki et al. 2008).

#### Necroptosis/Necrosis

When activation of caspases is prevented, DNA damage can induce an alternate form of cell death termed necroptosis. Necroptosis depends on hyperactivation of the poly-ADP-ribose-polymerase (PARP), a protein involved in DNA excision repair, and subsequent activation of receptor-interacting protein (RIP)—kinases as a response to depletion of intracellular ATP. Necroptosis, once being triggered by a structure called the necrosome, is characterized by the appearance of reactive oxygen species (ROS), lipid peroxidation, failure in calcium homeostasis, organelle swelling, and plasma membrane rupture (Vandenabeele et al. 2010). It appears to be of special importance in cancer cells of epithelial origin which reveal a limited apoptosis induction capacity in response to ionizing radiation, and also when irradiation is applied in high doses or in combination with hyperthermia (Mantel et al. 2010; Schildkopf et al. 2010). Additionally, high doses of ionizing radiation can stimulate necrosis, an accidental, uncontrolled type of cell death, which is predominantly characterized by rupture of the plasma membrane and a resulting release of intracellular contents, including danger signals, which can potently alert the immune system.

#### Mitotic catastrophe

The term “mitotic catastrophe” describes a cellular condition, which results from aberrant cell cycle progression prior to mitotic entry or during cell division itself. Mitotic catastrophe is characterized by the formation of huge cells with multiple nuclei as well as hyperamplified centrosomes. It might constitute the predominant mechanism of radiation-dependent cell death in cells with defective cell cycle checkpoints (Eriksson and Stigbrand 2010). However, cells, which have undergone mitotic catastrophe, might survive for several days, transit into senescence, or die by apoptosis and/or necro(pto)sis due to their high degrees of aneuploidy.

#### Cellular senescence

Cellular senescence is a state of permanent cell cycle arrest, which can be instigated by DNA damage. Senescence induction requires function of certain cell cycle checkpoint components, such as p53 and the retinoblastoma protein pRB, but it has also been observed in the absence of functional p53 (Nardella et al. 2011). Senescent cells are active in terms of metabolism, but do not show further cell cycle progression. Central features of senescent cells comprise a flattened morphology, an increase in granularity, the up-regulation of cyclin-dependent kinase inhibitors, and a positive staining for β-galactosidase (SA-β-Gal). Furthermore, senescent cells have been reported to release factors that can support as well as inhibit malignant progression by influencing both the proliferation of neighboring cells and antitumor immune responses (Krtolica et al. 2001; Eriksson and Stigbrand 2010; Coppe et al. 2010).

#### Autophagy

Autophagy represents a cellular state that is currently being discussed as both a mechanism of cell death and cell survival (Apel et al. 2009). It is characterized by the sequestration of proteins and/or organelles within huge autophagic vesicles called autophagosomes. As fusion of these vesicles with lysosomes leads to the formation of autophagolysosomes and degradation of their content providing material for de novo synthesis and regeneration, it is rather unclear whether autophagy represents a mechanism of survival or cell death, respectively. Autophagy involves the activation of multiple protein kinases, including the class I phosphatidylinositol-3-kinases (PI3 K-I), stress kinases, and the mammalian target of rapamycin (mTOR) kinase, and it has been observed in response to exposure to ionizing radiation (Apel et al. 2008).

#### Immunological consequences

The induction of tumor cell death and the inhibition of clonogenic survival by the application of ionizing radiation are central elements of its therapeutic success. Yet, it is well accepted that mechanisms involving both the innate and the adaptive immune system contribute to tumor regression—particularly in the context of ablative radiotherapy, where irradiation is applied in high single doses of 10 Gy or more (Lauber et al. 2012). In this regard, local high-dose radiotherapy of transplanted mouse B16 melanoma has been reported to stimulate the generation of tumor antigen-specific, interferon-γ (IFN-γ)-producing T cells (Lugade et al. 2005). Moreover, ablative, but not fractionated, radiotherapy drastically enhanced T cell priming in tumor-draining lymph nodes, which was paralleled by a regression of the primary tumor as well as distant, out-of-field metastases in a CD8^+^ T cell-dependent manner (Lee et al. 2009). Mechanistically, these T cells apparently have been primed by dendritic cells (DCs), which carry ingested tumor material and cross-present it in the tumor-draining lymph nodes. A recent study showed that the intratumoral production of type I interferons (IFN-α/β) in response to ablative radiotherapy is key in this scenario, since it enhances the cross-presenting capacity of tumor-infiltrating DCs (Burnette et al. 2011). This cascade of interferons, where IFN-α/β produced by CD11c^+^ cells (presumably DCs and macrophages) enhances the cross-priming activity of CD8α^+^ DCs thereby stimulating the generation of IFN-γ-producing CD8^+^ T cells and, finally, tumor rejection, is well known from the field of tumor immunoediting (Diamond et al. 2011; Fuertes et al. 2013). Here, IFN-α/β and IFN-γ contribute on different levels to the reduction in tumor burden. Whereas IFN-α/β primarily exerts its effects on macrophages, DCs, and NK cells by facilitating their activation and maturation and by enhancing their capacity to induce adaptive immune responses (Dunn et al. 2006), IFN-γ directly affects the tumor via inhibition of tumor cell proliferation, apoptosis induction, inhibition of angiogenesis, and an overall enhancement of tumor immunogenicity (Dunn et al. 2006; Lugade et al. 2008; Reits et al. 2006). Additionally, IFN-γ contributes to the stimulation of an antitumor immune response since it is essentially involved in T_H_1/T_C_1 cell responses and exerts similar effects as IFN-α/β in terms of innate immune cell activation and DC-mediated antigen cross-presentation (Dunn et al. 2006). This interferon cascade of innate and adaptive immune responses has only been described in case of ablative but not conventional, fractionated radiotherapy (Lee et al. 2009), and the question that needs to be addressed is why. One feasible explanation could be that ablative and fractionated radiotherapy trigger different tumor cell responses in terms of cell death and/or senescence induction with only high single-dose irradiation stimulating primary or secondary, postapoptotic secondary necro(pto)sis or senescence, respectively. The corresponding cellular releasates, a complex mixture of danger signals, and the senescence-associated secretome are well known to be potent inducers of IFN-α/β and other pro-inflammatory cytokines and hence could initiate the IFN-cascade described above and the DC-mediated instigation of antitumor T cell responses (Coppe et al. 2010; Apetoh et al. 2007; Peter et al. 2010; Kuilman and Peeper 2009).

### Combination of radiotherapy (RTX) with targeted agents

**Fig. 3:**
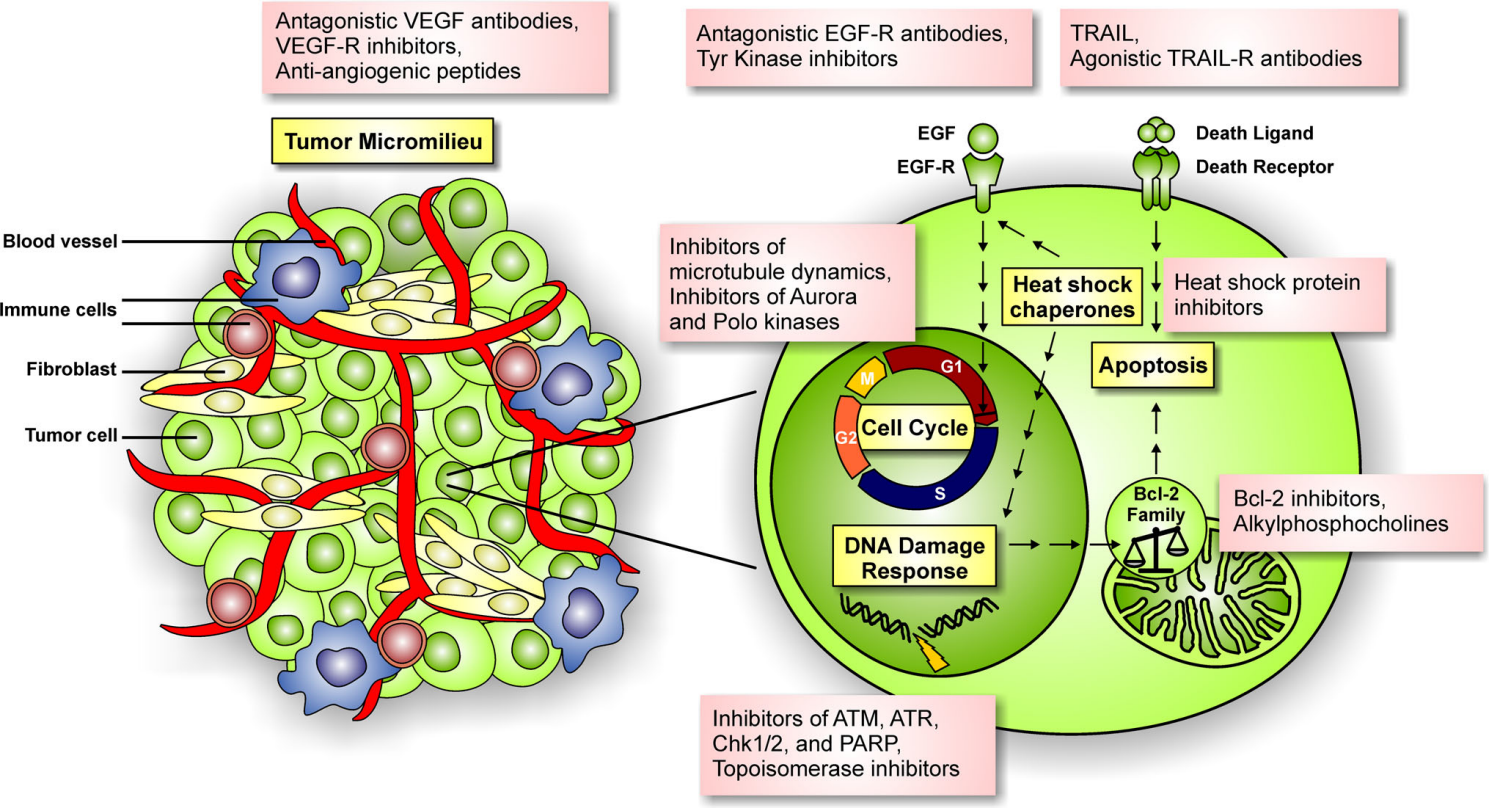
Survey of valuable targets for combined modality approaches

Despite the technical improvements in cancer radiotherapy in recent years, the combination of radiotherapy with classical chemotherapy has reached a dead end (Budach et al. 2006). Therefore, novel strategies encompassing the combination of conventional radiotherapy with agents that are specifically raised against key factors of malignant transformation have been designed and are currently being tested (Fig. 3). In the following paragraphs, current efforts made in order to specifically target cellular compounds to improve the efficacy of clinical radiation oncology in the future are discussed.

#### Combination of RTX with agents targeting the DDR

As the cell-death-inducing potential of ionizing radiation is largely determined by the cells’ capacity to cope with DNA damage, it is no wonder that both the expression and the functionality of DDR components have great impact on the efficacy of radiotherapy. This can be appreciated by the fact that the expression of DDR components within different tissues often correlates with the resistance or sensitivity of the respective tissue toward irradiation (Peters et al. 1982; Deacon et al. 1984). Therefore, targeted pharmaceutical agents, which interfere with proper function of the DDR, should be suitable to enhance the efficacy of conventional radiotherapy (Basu et al. 2012; Begg et al. 2011). Indeed, several studies revealed that interfering with ATM function (e.g., by using small-molecule inhibitors such as KU-55933) efficiently sensitizes human cancer cells to irradiation (Hickson et al. 2004; Cowell et al. 2005; Golding et al. 2009). Similar results have been reported for inhibitors of other DDR kinases, e.g., the ATR inhibitors VE-821 and VE-822 (Prevo et al. 2012; Pires et al. 2012; Fokas et al. 2012) or the CHK1/2 inhibitor AZD7762 (Zabludoff et al. 2008; Mitchell et al. 2010; Morgan et al. 2010). Other targets within the DDR network are the PARPs as this class of enzymes is involved in repair of DNA single-strand breaks (SSBs)—a kind of DNA damage commonly induced by ionizing radiation. Indeed, several PARP inhibitors, such as olaparib (AZD-2281) and veliparib (ABT-888), have revealed great potentials in terms of sensitizing tumor cells to irradiation in combined modality approaches (Donawho et al. 2007; Barazzuol et al. 2013; Miura et al. 2012; Chalmers et al. 2004; Senra et al. 2011; Shelton et al. 2013) and are, therefore, tested in clinical trials (Audeh et al. 2010; Tutt et al. 2010; Kaye et al. 2012). Recently, a specific inhibitor of the non-homologous end joining (NHEJ)-associated DNA ligase IV has been published (Srivastava et al. 2012) and showed great potency in terms of radiosensitizing cancer cells both in vitro and in vivo (Srivastava et al. 2012). Further studies are needed in order to see whether this inhibitor is feasible for clinical purposes.

Meanwhile, the small-molecule-inhibitor-based interference with DDR function might also offer the possibility to specifically target cancer stem cells (CSCs)—a small subset of the tumor cell population that shares several features with normal stem cells, e.g., the potential to self-renew, to proliferate excessively, to differentiate into multiple cellular lineages, and to induce de novo formation of blood vessels (Reya et al. 2001; Jordan et al. 2006). CSCs have moved into the focus of targeted therapies in recent years since complete eradication of a tumor inevitably demands for elimination of this particular kind of tumor cells that have the potential to self-renew and, in consequence, exhibit clonogenicity. Notably, CSCs exhibit an enormously high level of radioresistance (Bao et al. 2006; Firat et al. 2011), but the underlying mechanisms are unknown. It has been suggested that lower levels of ROS generated within the CSCs contribute to their high degree of radioresistance as well as to their enormous capacity to cope with DNA damages (Bao et al. 2006; Diehn et al. 2009). Very recently, it has been reported that CSCs exhibit a great enhancement in ATM kinase activity, suggesting that ATM might be a valuable target for combined modality approaches aiming at overcoming CSC radioresistance (Yin and Glass 2011). Indeed, Yin and Glass show that the inhibition of ATM by a small-molecule inhibitor reduces the radioresistance of CSCs (Yin and Glass 2011), thereby offering novel therapeutic perspectives. Aside, multiple signal transduction pathways that are important for the development of non-transformed stem cells, including the notch-, the hedgehog-, and the Wnt-/β-catenin pathway, have been reported to contribute to radioresistance in CSCs (Chen et al. 2007; Phillips et al. 2006; Woodward et al. 2007; Wesbuer et al. 2010; Cerdan and Bhatia 2010). This might offer additional prospects for combinatorial approaches in future.

#### Combination of RTX with agents targeting topoisomerases

Topoisomerases represent a class of enzymes that regulate the topology of DNA, e.g., during processes such as replication, transcription, recombination, and DNA repair. While topoisomerase I (Topo I) coordinates relaxation of superhelical DNA by introducing single-strand breaks (nicks) within the DNA duplex, topoisomerase II (Topo II) introduces transient double-strand breaks, thereby disentangling coiled DNA (Champoux 2001). As these functions are crucial both for the integrity and for the propagation of genomes, topoisomerases became one of the first classes of enzymes targeted in cancer therapy. Primarily, inhibitors that were derived from camptothecin (inhibits topoisomerase I) and etoposide/VP-16 (inhibits topoisomerase II) were deployed to target the function of topoisomerases. Aside from their immense chemotherapeutic potential per se, these drugs also turned out to possess an excellent potential in terms of sensitizing tumor cells toward ionizing radiation (Chen et al. 1999). In parallel, several synthetic analogues, such as topotecan and irinotecan, were raised and investigated for clinical purposes (Pommier 2013). The results obtained confirmed the notion that pharmaceutical inhibition of topoisomerases provides a good opportunity for combined modality treatment of multiple kinds of neoplasms (Mattern et al. 1991; Kim et al. 1992; Choy and MacRae 2001). This explains why respective combinations have been and still are enduringly tested within clinical trials (O’Leary and Muggia 1998; Hande 1998; Tao et al. 2013). In addition, multiple other classes of topoisomerase inhibitors, such as quinolines (inhibitors of topoisomerase I), quinolones, and anthracyclines (inhibitors of topoisomerase II), have been deployed for clinical purpose (Pommier 2013).

#### Combination of RTX with agents targeting the apoptosis network

As the induction of cell death—at least in part—depends on the functionality of the apoptotic machinery, drugs that can directly stimulate apoptosis (for instance, by facilitating caspase activation) also moved into the view of clinically oriented research, especially as it can be assumed that targeting of apoptotic network components should efficiently sensitize tumor cells toward ionizing radiation. Moreover, many kinds of tumor cells circumvent efficient induction of apoptosis by down-regulation of pro-apoptotic genes or up-regulation of antiapoptotic ones (Kasibhatla and Tseng 2003). One prominent target among these components is the TNF-α-related apoptosis-inducing ligand (TRAIL/Apo2L). For several tumor cell lines, it could be shown that both recombinant TRAIL itself and TRAIL-receptor agonistic antibodies, e.g., mapatumumab and lexatumumab, efficiently sensitize tumor cells to ionizing radiation (Belka et al. 2001; Chinnaiyan et al. 2000; Gong and Almasan 2000; Marini et al. 2009a, b; Niyazi et al. 2009a, b). In particular, cells that displayed only weak responses to either treatment alone often showed strong sensitization effects while no effect could be detected for non-transformed cells, which is—at least in part—due to the high level of selectivity of TRAIL and TRAIL-receptor agonizing antibodies for malignant cells. Another class of proteins involved in the regulation of apoptosis and therefore representing a promising target for combined modality approaches are the members of the B-cell lymphoma 2 (Bcl-2) family (Vogler et al. 2009). This protein family regulates the permeabilization of the outer mitochondrial membrane—a prerequisite for apoptosis induction via the intrinsic pathway. Therefore, inhibition of antiapoptotic Bcl-2 proteins should enhance the induction of apoptosis, especially when being combined with irradiation. In fact, several studies showed that inhibition of Bcl-2 sensitizes tumor cells toward ionizing radiation (Zerp et al. 2009; Moretti et al. 2010), revealing that Bcl-2 and, possibly, other members of this protein family may serve as candidates for targeted approaches in the future. Currently, navitoclax (ABT-263), a highly selective Bcl-2 inhibitor, is tested in clinical trials, and initial results strengthen the hope for its future implementation in the clinic (Gandhi et al. 2011; Rudin et al. 2012). A third class of compounds known to promote both intrinsic activation of apoptosis and radiosensitization are phospholipid analogues, such as the membrane-targeted alkylphosphocholines miltefosine and perifosine (Hilgard et al. 1997; Unger et al. 1989). The radiosensitizing capacity of this kind of drugs has already been proven in multiple tumor entities (Gao et al. 2011; Henke et al. 2012; Vink et al. 2006, 2007; Berkovic et al. 1997; Ruiter et al. 1999; Rubel et al. 2006).

#### Combination of RTX with agents targeting cell division

The cell cycle phase in which cell division takes place (M-phase) is considered to be the most vulnerable state in terms of radiotherapeutic intervention as it is well acknowledged that the sensitivity of cells to ionizing radiation peaks at this cell cycle stage (Sinclair and Morton 1966; Terasima and Tolmach 1963). Therefore and because of the fact that tumor cells, in contrast to most other non-transformed cell types, divide extensively, ancient approaches already aimed at arresting tumor cells within M-phase in order to achieve a maximum in radiosensitivity. For this purpose, drugs mainly derived from natural origin, such as taxol/paclitaxel, colchicine, and colcemid, were initially used. These compounds interfere with microtubule dynamics, thereby preventing accurate execution of cell division which results in a permanent arrest of the cells within M-phase. As to be expected, several of these drugs exhibited synergistic effects when being combined with exposure to ionizing radiation (Griem and Malkinson 1966; Brues et al. 1940; Tishler et al. 1992; Milas et al. 1994, 1996; Milross et al. 1997). This is why some of them (e.g., taxol) not only are adopted in radiochemotherapy but even still are in the focus of current clinical research (Pergolizzi et al. 2011; Combs et al. 2012). However, these drugs not only lack the level of specificity current therapies demand for, but they also exhibit side effects, which, in worst case, even limit the therapeutic effort. Progression through M-phase and the process of cell division itself both depend on the function of a multitude of cellular proteins including many protein kinases, which offers great opportunities for pharmaceutical intervention. In recent years, small-molecule inhibitors targeting protein kinases, which function more or less exclusively during cell division (e.g., Aurora kinases and Polo-like kinases), were designed and tested for their utility in combinatorial approaches. In these studies, several compounds, e.g., the Aurora kinase inhibitors AZD1152 (Barasertib), VX-680 (Tozasertib), and MLN8054, as well as the Polo-like kinase-1 inhibitor BI2536, have proven radiosensitizing potential (Moretti et al. 2011; Tao et al. 2008, 2009; Guan et al. 2007; Harris et al. 2012), nourishing the hope for their future implementation in the clinic.

#### Combination of RTX with agents targeting the heat shock response

Heat shock proteins (HSPs) are molecular chaperones that catalyze the proper folding of other proteins and thereby avoid protein aggregations within cells. HSPs are often overexpressed in tumor cells as these cells are characterized by an overall increased level of protein synthesis, thus necessitating effective chaperone function in order to prevent misfolding and/or aggregation of proteins in these cells. In addition, HSP expression can be induced in response to multiple physiological or environmental insults, including irradiation, hypoxia, and/or chemical stress (Young et al. 2004). In this context, HSPs frequently function in an antiapoptotic fashion by associating with key components of the apoptotic machinery, thereby interfering with efficient apoptosis induction. For example, HSP70 and HSP90 can interfere with caspase-dependent and caspase-independent apoptosis induction as well as by binding to the pro-apoptotic proteins Apaf-1 and apoptosis-inducing factor (AIF) (Garrido et al. 2006).

These findings explain why compounds that obstruct HSP function came into the focus of clinical research in recent years. Initially, naturally derived inhibitors targeting HSPs, such as geldanamycin and radicicol, were tested for clinical purposes but turned out to exhibit fatal side effects such as liver toxicity, thus precluding their implementation in the clinic. Therefore, novel compounds have been designed molecularly in order to minimize these kinds of side effects concomitant with a maximum in HSP-inhibiting capacity (Chiosis et al. 2006). Among those, inhibitors of HSP90 such as 17-N-allylamino-17-demethoxygeldanamycin (17-AAG), 17-dimethylaminoethylamino-17-demethoxygeldanamycin (17-DMAG), or NVP-AUY922, in particular, exhibited convincing potential in promoting tumor cell death as well as in sensitizing tumor cells to ionizing radiation (Bisht et al. 2003; Bull et al. 2004; Russell et al. 2003; Machida et al. 2005; Matsumoto et al. 2005; Kabakov et al. 2008; Stingl et al. 2010; Milanovi et al. 2013).

#### Combination of RTX with agents targeting the EGFR pathway

Another promising target for combined modality approaches is the EGFR, one member of the epithelial tyrosine kinase-associated membrane receptor family, and its downstream signaling pathways (Davies et al. 1980). Activation of EGFR leads to cell proliferation, inhibition of apoptosis, and angiogenesis. EGFR expression is commonly increased in human cancers (Wernicke et al. 2010), and preclinical evidence suggests a direct impact of EGFR on the sensitivity of tumor cells toward ionizing radiation (Milas et al. 2000; Akimoto et al. 1999). In accordance, the expression of EGFR was reported to be up-regulated in response to irradiation, which might attenuate the effectiveness of fractionated radiotherapy (Fedrigo et al. 2011). Indeed, overexpression as well as mutations in the EGFR gene was shown to directly correlate with tumor radioresistance and poor clinical prognosis (Lammering et al. 2004; Giralt et al. 2005). Therefore, the EGFR pathway exhibits great influence on the overall effect that can be achieved by clinical irradiation, which in turn offers great opportunities for pharmaceutical intervention. Various kinds of EGFR-inhibiting molecules, such as the monoclonal antibodies cetuximab and panitumumab as well as the tyrosine kinase inhibitors erlotinib and gefitinib, have been developed and demonstrated great therapeutic benefit both in preclinical reports and in randomized clinical trials when combined with ionizing radiation. Therefore, EGFR inhibition meanwhile has become an established part of the clinical routine in radiation oncology (Nieder et al. 2012).

#### Combination of RTX with agents targeting the tumor micromilieu

Solid tumors are usually composed of tumor cells and several other cell types that form the tumor micromilieu. Both the formation and the progression of a solid tumor depend on the tight interaction between transformed tumor cells and the cells in the tumor microenvironment. By secreting growth factors and cytokines that target endothelial cells, fibroblasts, and other cell types within the microenvironment, tumor cells actively shape their surrounding milieu, for instance by inducing de novo formation of blood vessels and extracellular matrices (Carmeliet and Jain 2011). Moreover, tumor cells can also acquire the capacity to skew or evade antitumor immune responses and even to induce a milieu of immune tolerance (Dunn et al. 2004).

The complex interplay between tumor cells and the tumor stroma has strong impact on the tumor’s sensitivity to exposure to ionizing radiation and, therefore, on long-term tumor control following radiotherapeutic attendance. In this respect, understanding the effects of ionizing radiation on the tumor microenvironment rather than on isolated tumor cells is one of the greatest interests in current radiobiological science. One promising candidate for radiotherapeutic approaches is the tumor microvasculature (Garcia-Barros et al. 2003). Recent reports suggest that directly targeting angiogenesis might increase the therapeutic ratio when being combined with irradiation (Beal et al. 2011). In accordance, the monoclonal antibody bevacizumab, which blocks angiogenesis by preventing the binding of VEGF to its respective receptor (Willett et al. 2004), significantly improves clinical outcome when combined with radiotherapy (Velenik et al. 2011; Shin et al. 2011; Niyazi et al. 2012a), and similar results were obtained for the VEGF-R inhibitor vandetanib and, primarily, for the antiangiogenetic peptide cilengitide (Albert et al. 2006; Williams et al. 2004; Brazelle et al. 2006; Drappatz et al. 2010; Yang et al. 2010). However, a recent phase III trial on cilengitide in combination with radiochemotherapy failed to show a significant increase in overall survival in glioblastoma patients.

Another mediator of the microenvironment’s response to irradiation is transforming growth factor-β (TGF-β), which is activated in response to ROS (Barcellos-Hoff and Dix 1996). TGF-β regulates the proliferation, the differentiation, and the migration of cells (Massagué et al. 2000) and also contributes to metastasis and cell invasion (Heldin et al. 2009; Pardali and Moustakas 2007). This explains why interfering with TGF-β signaling may decrease tumor cell growth, as well as their motility and their metastasizing capacity (Ikushima and Miyazono 2010). Thus, inhibition of TGF-β can actively modulate the tumors’ response to ionizing radiation, thereby providing an interesting tool for combinatorial approaches (Flanders and Burmester 2003; Rabbani et al. 2003; Xavier et al. 2004).

## Side effects

As exposure to ionizing radiation induces cell death, radiotherapy inevitably coincides with side effects, including degeneration of normal tissues, acute inflammation, and even fibrotic tissue remodeling. The implementation of modern techniques such as IMRT has greatly facilitated the reduction in these classical kinds of side effects. On the other hand, novel, combined modality approaches that employ novel, molecularly designed compounds have led to rise of new, so far unknown side effects.

### Classical side effects

Both acute inflammation and chronic fibrosis are classical side effects that coincide with the radiotherapeutic treatment of neoplasms and may limit radiation doses and thus the efficacy of the treatment (Abratt et al. 2004; Plathow et al. 2004; Abdollahi et al. 2005). In some cases, e.g., lung cancer, dose limitations due to the restricted tolerance of normal tissues even preclude successful radiotherapy in many patients with advanced disease progression (McDonald et al. 1995; Rosenzweig et al. 2000). In general, the severity of irradiation-induced pneumonitis depends on treatment factors, such as totality of the dose, the volume of irradiated lung, the schedule of fractionation, and the chemotherapy administered (Taghian et al. 2001; Rosen et al. 2001; Shi et al. 2010; Blom Goldman et al. 2010), but also on patient- and/or disease-related factors, such as preexisting lung diseases, poor pulmonary function, or genetic predispositions (Movsas et al. 1997; Mertens et al. 2002; Abratt et al. 2004). However, the mechanisms underlying these side effects are still poorly understood.

Although irradiation-induced primary damages in target cells such as apoptosis and necrosis have been sufficiently documented (Eriksson and Stigbrand 2010; McBride 1995), subsequent biological reactions in irradiated organs are quite sophisticated and not well defined (Lindroos et al. 1995; Zhang and Phan 1996). Recent studies suggest that cytokine cascades that govern the signaling pathways involved in irradiation response may play a pivotal role within these processes (Pohlers et al. 2009; Li et al. 2007; Lee et al. 2010), and a growing body of evidence demonstrates an increased expression of cytokines in radiation-induced pulmonary lesions (Johnston et al. 1996; Abdollahi et al. 2005). Among these, some pro-inflammatory cytokines such as the TNF- and the CD95 ligands are of importance for acute inflammation (Johnston et al. 1996; Heinzelmann et al. 2006), while others, such as TGF-β and PDGF, are more involved in the regulation of chronic fibrotic response (Abdollahi et al. 2005; Dancea et al. 2009).

Recently described strategies that directly interfere with intracellular signaling pathways have revealed encouraging results in terms of attenuating radiation-caused side effects (Abdollahi et al. 2005; Anscher et al. 2008; Puthawala et al. 2008). However, as the cytokine signaling pathways that are activated in response to irradiation are broadly overlapping, rather than being independent of each other, it is unlikely that a complete blockage of these reactions can be achieved by blocking only one of them (Li et al. 2009; Wynn 2008). Thus, multitargeted agents should exhibit higher effectiveness in attenuation of radiation-induced inflammation and fibrogenesis.

### Novel side effects due to employment of targeted agents

With the increase in clinical relevance of novel, molecularly targeted agents, novel kinds of side effects are emerging (Niyazi et al. 2011b). Unfortunately, clinical data that would allow the assessment of these side effects are scarce. Additionally, the heterogeneity of both targeted agents and study designs does not allow abstraction these side effects. The examples presented here are meant to give an insight into the wide variety of side effects that may arise due to employment of targeted agents.

On the one hand, huge clinical trials exist for targeted agents such as trastuzumab, a humanized monoclonal anti-her-2/neu antibody approved for the treatment of her-2/neu-positive breast cancers, showing no significant additional effects if being combined with radiation in a short-time follow-up (Halyard et al. 2009). On the other hand, there are agents such as sorafenib or erlotinib belonging to the group of kinase inhibitors for which toxicity data upon combined usage are extremely rare. However, case reports exist, in which combinational or sequential application of radiotherapy and kinase inhibitors were shown to lead to severe or even fatal toxicities such as diarrhea (Silvano et al. 2008), bowel perforation (Peters et al. 2008), and bronchial fistula (Basille et al. 2010).

The most prominent and rather well-documented example of a non-classical side effect can be observed for the EGFR-antagonizing antibody cetuximab which, for example, has been successfully used in combination with radiotherapy for the treatment of head and neck cancers (Bonner et al. 2006). In the trial conducted by Bonner and colleagues, a significant improvement in overall survival of patients that were treated with radioimmunotherapy was observed when compared to patients treated with radiotherapy alone. During this trial, the combinational treatment was reported to be rather well tolerated; however, during the years of clinical use, multiple reports pointing out an increase in skin toxicity and cases of even severe skin toxicity have been published (Walsh et al. 2011; Koutcher et al. 2009; Giro et al. 2009; Berger and Belka 2008).

Another targeted agent that exemplifies the heterogeneity of putative toxicities is the VEGF-antagonizing antibody bevacizumab that is used in combination with radiotherapy in different anatomical regions. Promising attempts were made in the combination of radiotherapy with bevacizumab for the treatment of (recurrent) glioblastomas (Beal et al. 2011; Vredenburgh et al. 2012). While no increased infield bleeding was reported for the application of ionizing radiation to the CNS, some cases of wound dehiscence of the previously operated site as well as increased levels of toxicity at late stages with some cases of optic neuropathy and one single case of Brown–Séquard syndrome have been documented (Gutin et al. 2009; Niyazi et al. 2012a; Lai et al. 2008; Kelly et al. 2011). Concerning the combination of bevacizumab and radiotherapy in case of the gastrointestinal tract, some studies pointed out an increased toxicity level, such as ischemic bowel complications (Lordick et al. 2006), mucosal tumor-associated bleeding (Crane et al. 2010), GI-bleeding, ulceration (Crane et al. 2010), and wound complications (Dipetrillo et al. 2012). Finally, in case of the mediastinal region, an increased rate of tracheoesophageal fistula has been reported (Spigel et al. 2010).

## Prognosis and prediction

To date, therapeutic decisions are taken on increasing individualized and personalized bases. Important criteria in this regard are markers that help to predict the overall prognosis of the patient, the potential success of a particular kind of therapy, and the occurrence of unwanted side effects. In particular, the combination of ionizing radiation with molecularly targeted agents requires an a priori identification of patients that will benefit most (or at all) from a respective therapy. Here, classical parameters such as age, tumor node metastasis (TNM) stage, and histology of the tumor might not be sufficient, and additional information concerning the molecular tumor characteristics is needed in order to find the best therapeutic approach for the individual patient. “Prognostic” markers, in general, provide information concerning the natural course of the respective disease independently of the treatment applied. In contrast, the term “predictive” refers to markers for which it is likely that a specific subgroup among the patient collective will benefit from a certain intervention. For example, the EGFR1 mutation has a predictive value in adeno-NSCLC patients, but not a prognostic one (Oldenhuis et al. 2008).

### Biomarkers for tumors

In patients with malignant gliomas, it should be of standard to test for the mutational status of the genes encoding for isocitrate dehydrogenases 1 and 2 (IDH-1/-2) as well as for codeletion of the 1p/19q loci. While mutations within the IDH-1/-2 genes can be found in more than 70 % of all primary astrocytomas (WHO grades II/III), oligodendrogliomas, and secondary glioblastomas, the respective mutation rate is only about 5 % in primary glioblastomas and mutations within IDH-1/-2 are associated with positive clinical prognosis in astrocytoma and glioblastoma (Yan et al. 2009; Combs et al. 2011). In parallel, the codeletion of 1p/19q was shown to correlate with reduced tumor aggressiveness and better response in anaplastic oligodendroglioma (Cairncross et al. 2006; Quon and Abdulkarim 2008; van den Bent et al. 2006). In addition, also the methylation status of the O-(6)-methylguanine-DNA methyltransferase (MGMT) gene promoter should be investigated. MGMT is a DNA-repairing enzyme that decreases the effects achievable by alkylating agent (e.g., temozolomide)-based chemotherapy (Esteller et al. 2000). Temozolomide is routinely used for concomitant radiochemotherapy in malignant gliomas as it was shown that combining temozolomide with radiotherapy results in significant prolongation of patient survival (Hegi et al. 2005; Stupp et al. 2009). As methylation of the MGMT promoter represses the expression of MGMT, this leads to a better response and thus, the methylation status of the MGMT promoter should be tested in routine before starting a temozolomide-based therapy.

Carcinogenesis in squamous cell carcinomas of the head and neck (HNSCC) can be linked either to the frequent use of tobacco and alcohol or to human papillomavirus (HPV) infection. In HPV-positive tumors, p53 and pRB tumor-suppressor function is blocked by viral proteins called E6 and E7, respectively, culminating in high levels of genome instability and increased expression of the senescence-associated Cdk1-inhibitor p16^Ink4a^. Detection of the HPV status can be accomplished by real-time PCR, and p16^Ink4a^ can be detected by immunohistochemistry (Snow and Laudadio 2010). Approximately one-quarter of all HNSCC patients are positive for HPV (Deacon et al. 1984), and in oropharyngeal carcinomas, the prevalence of a positive HPV status is even around 40 %. Moreover, HPV-positive tumors not only genetically differ from negative ones (Martinez et al. 2007), but they also differ in terms of capacity to cope with DNA damage which is reduced in the HPV-positive tumors (Rieckmann et al. 2013). This can also explain, at least in part, why the HPV status is such an important prognostic factor in HNSCC patients, as it is often associated with superior outcome in case of patients treated with surgery followed by adjuvant radiotherapy or definitive radiochemotherapy (Ihloff et al. 2010; Fischer et al. 2010; Prestwich et al. 2010).

### Biomarkers for side effects

One limitation in the radiotherapeutic treatment for malignant tumors is given by the need to minimize toxic effects that may harm normal tissues. In this context, late complications are of special importance because of frequently showing progression and thus association with long-life risk (Jung et al. 2001). Meanwhile, the extent of tissue toxicity introduced by irradiation greatly varies among different patients. Even though inherited hypersensitivity syndromes such as ataxia telangiectasia and the Nijmegen breakage syndrome that are characterized by severe side effects are rare, a wide range of reactions within normal tissues can be detected among the standard population. It was suggested that such individual variations in radiosensitivity are caused by genetic differences, such as single nucleotide polymorphisms (SNPs) (Turesson et al. 1996; Safwat et al. 2002). As these may serve as markers that would allow for estimating the individual risk of radiation-induced toxicity to non-transformed tissues, extensive efforts were made to identify such markers. Indeed, several SNPs could be identified that show tight relation with the degree of radiotoxicity as exemplified by SNPs that reside in the IL12RB2 and the ABCA1 genes (Isomura et al. 2008) as well as within the ATM gene (Edvardsen et al. 2007; Xiong et al. 2012). However, the studies performed so far often give rise to heterogeneous and/or even conflicting results. This can be seen for instance by the C-509 T polymorphism, an extensively studied SNP of the TGFβ1-encoding gene, for which conflicting results have been reported regarding its role in promoting inflammatory and fibrotic effects (Quarmby et al. 2003; Andreassen et al. 2005; De Ruyck et al. 2006; Barnett et al. 2010; Martin et al. 2010). Moreover, it was shown that significant coincidence of SNP occurrence and tissue toxicity is only found when several SNPs and/or other risk alleles are combined (Alsner et al. 2008; Andreassen et al. 2006; Zschenker et al. 2010). However, these data also have been contradicted by other studies (Raabe et al. 2012; Barnett et al. 2012). Therefore, analyzing the presence of SNPs as biomarkers that allow for individual prediction of side effects is still far from routine.

### Personalized medicine: imaging for prognosis and prediction

[^18^F]FDG-PET imaging has become the standard in oncologic treatment over the recent years especially for staging purposes due to its higher sensitivity and specificity if being compared to conventional imaging modalities such as CT and MRI. PET tracers may serve as prognostic and predictive markers for estimating responsiveness to radiotherapy or combined radiochemotherapy (Bussink et al. 2011). The outcome of head and neck cancer patients has been related to standardized uptake value (SUV) changes in PET imaging (Allal et al. 2004). Several tumor entities have been described in which PET gives early information as a marker for pathological response, especially in the cases of rectal cancer (de Geus-Oei et al. 2009), NSCLC (Pottgen et al. 2006), and esophageal cancer (Song et al. 2005). PET-CT was even described to be complimentary to conventional CT scan and able to predict early recurrences in breast cancer (Evangelista et al. 2011). Ongoing Hodgkin trials are in part based on PET imaging, and the stratification in these trials is done according to PET positivity after several chemotherapy cycles; however, this has still to be regarded as an experimental concept. Involved-node radiotherapy has been proposed as a means to further improve the therapeutic ratio by reduction of radiation-induced toxicity (Kobe et al. 2010) substantially based on proper PET/CT staging. Altogether, PET seems to be a substantial part of personalized medicine providing prognostic information and enabling the clinician to base treatment strategies on this information.

Meanwhile, PET-CT has gained an important place in radiotherapy planning (Yaromina and Zips 2010) as it provides detailed information about the tumor microenvironment in addition to anatomical imaging. In first instance, PET imaging data can be used for better delineation of the target volume. A second strategy, dose painting by contours (DPBC), consists in the creation of an additional PET-based target volume that is then treated with higher dose levels. In contrast, dose painting by numbers (DPBN) aims for a local variation in dose prescription according to the variation in the PET signal (Thorwarth et al. 2010). For instance, in case of lung cancer, several approaches already are available that directly depend on PET imaging (De Ruysscher et al. 2012). Currently, ^11^C-choline and occasionally ^18^F- or ^11^C-acetate are used as tracers for prostate cancer, reflecting the phospholipid metabolism (Pinkawa et al. 2011). ^11^C-choline-PET/CT might be considered as the imaging modality in radiation oncology to select and to delineate target volumes extending the prostate gland or fossa. In conjunction with IMRT and IGRT, it therefore might offer the opportunity for a dose escalation to selected sites while avoiding the irradiation of healthy tissues (Wurschmidt et al. 2011), and although the underlying assumption that PET correlates positively with more resistant subvolumes is still not proven for the broad variety of cancer types, data are coming forward that this is the case, e.g., in lung cancer. One open question is whether selective boosting with limited sensitivity of choline-PET indeed leads to higher tumor control rates (Niyazi et al. 2010).

Several trials are on their way to test PET imaging prospectively, e.g., in lung cancer [PET-PLAN trial (Fleckenstein et al. 2011)]. For malignant gliomas, FET-PET has been shown to significantly alter the target volumes (Niyazi et al. 2011a; Walter et al. 2012) and amino acid-PET in general, including 11C-methionine (MET)-PET, which was shown to be effective in target volume delineation (Grosu et al. 2005). The observation that meningioma cells overexpress the somatostatin receptor 2 (SSTR2) was the rationale to retrospectively analyze how far DOTATOC-PET/CT is helpful to improve target volume delineation for IMRT (Gehler et al. 2009). Many other tumor types are currently under investigation as PET provides additional information on tumor extent, involvement of lymph nodes, and putative distant metastases. Nevertheless, several problems have to be solved in the future, such as the inclusion of dynamic analyses and the correct procedures for thresholds.

## Conclusions

Radiotherapy represents a crucial treatment option in the treatment for malignant diseases. In the recent years, the efficacy of radiotherapy has been improved by new techniques, among which IMRT and IGRT may constitute the most important ones. In parallel, novel approaches that combine radiotherapy with molecularly designed agents specifically targeting the hallmarks of cancer have been deployed and revealed promising results both in preclinical models and in clinical trials. However, employment of such targeted agents often coincides with new kinds of side effects demanding for biomarkers, which allow for detailed patient stratification. As the current availability of such markers is far from satisfying, efforts to identify novel candidates must be increased. In parallel, research focusing on multimodality approaches must be intensified as conventional radiochemotherapy has reached its limits.
